# The reproductive tracts of two malaria vectors are populated by a core microbiome and by gender- and swarm-enriched microbial biomarkers

**DOI:** 10.1038/srep24207

**Published:** 2016-04-18

**Authors:** Nicola Segata, Francesco Baldini, Julien Pompon, Wendy S. Garrett, Duy Tin Truong, Roch K. Dabiré, Abdoulaye Diabaté, Elena A. Levashina, Flaminia Catteruccia

**Affiliations:** 1 Centre for Integrative Biology, University of Trento, Trento, Italy; 2 Department of Immunology and Infectious Diseases, Harvard T.H. Chan School of Public Health, Boston, MA 02115, USA; 3 Institute of Biodiversity, Animal Health and Comparative Medicine, University of Glasgow, Glasgow G12 8QQ, United Kingdom; 4 Dipartimento di Medicina Sperimentale e Scienze Biochimiche, Università degli Studi di Perugia, 06100 Italy; 5 UPR9022 CNRS, U963 Inserm, Université de Strasbourg, 15 rue R. Descartes, 67084 Strasbourg, France; 6 MIVEGEC (Maladies Infectieuses et vecteurs: écologie, génétique, évolution et controle), UMR IRD-CNRS-UM1-UM2, 34394 Montpellier, France; 7 Programme in Emerging Infectious Diseases, Duke-NUS Graduate Medical School, Singapore 169857; 8 Department of Genetics and Complex Diseases, Harvard T.H. Chan School of Public Health, Boston, MA 02115, USA; 9 Broad Institute of Harvard and MIT, Cambridge, MA 02142, USA; 10 Department of Medical Oncology, Dana-Farber Cancer Institute and Harvard Medical School Boston, MA 02115, USA; 11 Institut de Recherche en Sciences de la Santé/Centre Muraz, Bobo-Dioulasso, Burkina Faso; 12 Vector Biology Unit, Max-Planck Institute for Infection Biology, Chariteplatz 1, 10117 Berlin, Germany

## Abstract

Microbes play key roles in shaping the physiology of insects and can influence behavior, reproduction and susceptibility to pathogens. In Sub-Saharan Africa, two major malaria vectors, *Anopheles gambiae* and *An. coluzzii*, breed in distinct larval habitats characterized by different microorganisms that might affect their adult physiology and possibly *Plasmodium* transmission. We analyzed the reproductive microbiomes of male and female *An. gambiae* and *An. coluzzii* couples collected from natural mating swarms in Burkina Faso. 16S rRNA sequencing on dissected tissues revealed that the reproductive tracts harbor a complex microbiome characterized by a large core group of bacteria shared by both species and all reproductive tissues. Interestingly, we detected a significant enrichment of several gender-associated microbial biomarkers in specific tissues, and surprisingly, similar classes of bacteria in males captured from one mating swarm, suggesting that these males originated from the same larval breeding site. Finally, we identified several endosymbiotic bacteria, including *Spiroplasma*, which have the ability to manipulate insect reproductive success. Our study provides a comprehensive analysis of the reproductive microbiome of important human disease vectors, and identifies a panel of core and endosymbiotic bacteria that can be potentially exploited to interfere with the transmission of malaria parasites by the *Anopheles* mosquito.

The interplay between hosts and their endogenous microbiota is crucial for the physiology of a vast number of organisms ranging from mammals to plants^1^. In insects, the resident microbiome regulates nutrition, digestion, metabolism, reproduction, longevity and immunity^2^,^3^. The insect microbiota participate in the synthesis of essential nutrients that are scarce or unavailable in the host diet^4^, the production of enzymes increasing digestion efficiency^5^, and the provision of vitamins^6^. Microorganisms can also detoxify harmful chemicals such as flavonoids, tannins, and alkaloids present in plants, enabling the survival of many insects^7^.

In insect vectors of human diseases, the study of the microbiome holds tremendous potential for the development of microbial-based strategies to control vector-borne pathogens^8^,^9^. Prior microbiome studies of the *Anopheles* species that transmit malaria have focused on the analysis of the female midgut, the first tissue where *Plasmodium* parasites establish infection. *Anopheles* gut symbiotic bacteria dramatically influence sporogonic development of the most deadly malaria parasite, *Plasmodium falciparum*^10^,^11^,^12^,^13^,^14^, by either stimulating the host immune response^13^ or via the generation of damaging free radicals^14^. Interactions between *Anopheles* and the gut microbiota are complex as some bacteria interfere with parasite development^12^, while antibiotic-mediated perturbation of gut bacteria can increase *P. falciparum* infection rates^15^. Resistance to *Plasmodium* has also been genetically engineered using bacterial symbionts secreting anti-parasite molecules in the gut^16^, an approach known as paratransgenesis (reviewed in^17^).

In contrast with the evolving literature on *Anopheles* gut microbiota, the microbiome associated with insect reproductive organs has been largely unexplored, aside from studies focusing on *Asaia* and *Wolbachia*. *Wolbachia*, a maternally transmitted intracelullar bacterium residing in the ovaries and testes of nearly 60% of insect species, influences host reproductive success, immunity and lifespan, and can limit the development of vector-borne pathogens such as dengue virus and *Plasmodium*^18^,^19^. Recently, maternally transmitted *Wolbachia* infections were found in natural populations of *An. gambiae* and *An. coluzzii*, opening novel possibilities for malaria control^20^. *Asaia*, a bacterial genus populating both the male and female reproductive tracts of *Anopheles* as well as the gut^21^,^22^,^23^, has been proposed for paratransgenesis strategies given its ability to be paternally and maternally transmitted^21^,^22^,^23^.

Besides their role in pathogen resistance, reproductive tissue microbes may affect reproductive fitness and mating preferences. In *Drosophila melanogaster*, assortative mating was associated with symbiotic bacteria via the microbiota’s effects on cuticle mating pheromone synthesis^24^. Assortative mating behavior has been observed between *An. gambiae* and *An. coluzzii*, two closely related species (previously named S and M molecular forms, respectively) that live in sympatry over large areas in Sub-Saharan Africa but are only occasionally found to swarm together and form hybrids at very low frequencies^25^,^26^,^27^. These species are adapted to largely diverse larval habitats, with *An. coluzzii* using permanent breeding grounds like rice fields and *An. gambiae* predominantly found in temporary breeding sites^28^. Besides possible genetically-determined differences^29^ in cuticular hydrocarbon profiles^30^ and other mechanisms for reproductive isolation such as divergence in wing beat frequencies^31^, distinctive adult reproductive tract microbiomes fostered by different larval habitats may have favored the emergence of pre-mating barriers between these species, explaining the low prevalence of hybrids found in natural populations.

Such largely dissimilar habitats occupied during larval development may foster distinctive adult reproductive tract microbiomes, possibly creating pre-mating barriers and explaining the low prevalence of hybrids found in natural populations.

Microbiome surveys of the anopheline reproductive tracts have not been previously reported, despite the potential relevance for both reproductive success and mating ecology. Here we performed 16S rRNA amplicon sequencing and DNAseq investigations of the microbiomes populating male and female reproductive tissues of *An. gambiae* and *An. coluzzii* mosquitoes collected from natural mating swarms. Our results reveal that these two species share a highly abundant core microbiome present in both male and female tissues that could be exploited for paratransgenesis-based control strategies. Moreover, identification of swarm-specific microbial biomarkers suggests a spatial connection between larval breeding sites and swarm locations and provides unexpected insights into the mating biology of these important malaria vectors.

## Results

### 16S rRNA amplicon sequencing of reproductive tissues from *Anopheles gambiae* and *An. coluzzii* mating couples

**Figure 1:**
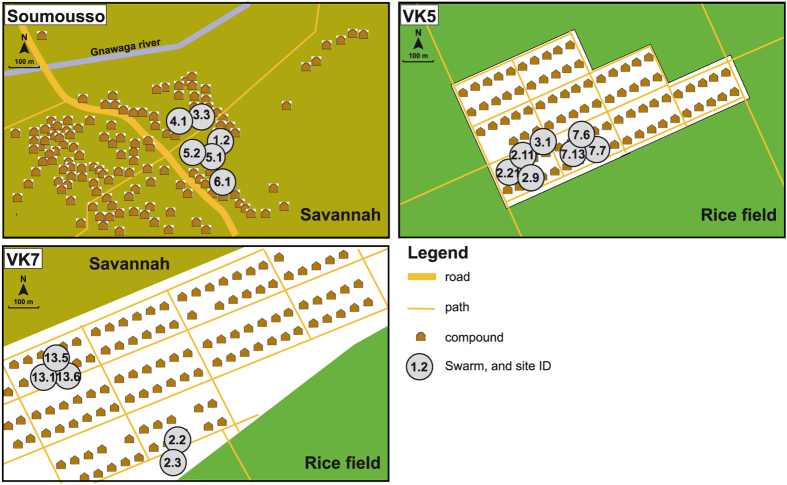
Schematic description of villages and mosquito swarm sites. The maps describe the three villages (Soumousso, VK5 and VK7) where *An. gambiae* and *An. coluzzii* couples were collected from mating swarms. Swarm sites are identified by numbers. Maps were modified from Fig. 1 published in^20^, originally adapted from A.A. Millogo, IRSS/Muraz.

To characterize the reproductive tract microbiomes of *An. gambiae* and *An. coluzzii* adults, mating couples were collected in natural swarms in three villages near Bobo-Dioulasso, Burkina Faso: Vallée du Kou 5 (VK5) and Vallée du Kou 7 (VK7), highly populated by *An. coluzzii*, and Soumousso, where *An. gambiae* are predominant (see Fig. 1 and Supplementary Table S1). Four reproductive tissues were isolated: the testes and male accessory glands (MAGs) from males, and the ovaries and lower reproductive tract (LRT, which comprises the atrium, the spermatheca and the parovarium) from females. We collected mating couples rather than resting males and females as we wanted to specifically study sexually active individuals. While the vast majority of collections (26 out of 30 mating couples) were composed of conspecific couples, we found 4 mixed *An. gambiae*/*An. coluzzii* couples, providing a 13.3% frequency of interspecific matings similar to previously reported hybridization frequencies in this geographical area^25^.

Samples were subjected to 16S rRNA amplicon sequencing (variable region V4, see Methods), and sequences were processed with diversity, taxonomic, and functional profiling pipelines^32^,^33^ to characterize the microbiome structure, composition, and variability. A number of samples (18) failed the analysis, providing a final number of 102 tissues successfully sequenced (Supplementary Table S2).

### The male and female reproductive tracts share a large core microbiome

We detected a high intra-sample diversity (i.e. alpha-diversity, defined as the number of different organisms, Supplementary Fig. S1) in the microbiome of the four reproductive tissues analyzed, with no clear differences driven by gender or species. At a subsampling rate of 3,500 sequences, we estimated a number of species-level sequence clusters (Operational Taxonomic Units, OTUs) in each sample ranging from 240 to 763 (average 500 s.d. 96). This overall high diversity was driven by a large number of OTUs present at low prevalence (i.e. fraction of positive samples) but high relative abundance (i.e. fraction of reads belonging to a given OTU in a sample, Supplementary Fig. S2).

**Figure 2:**
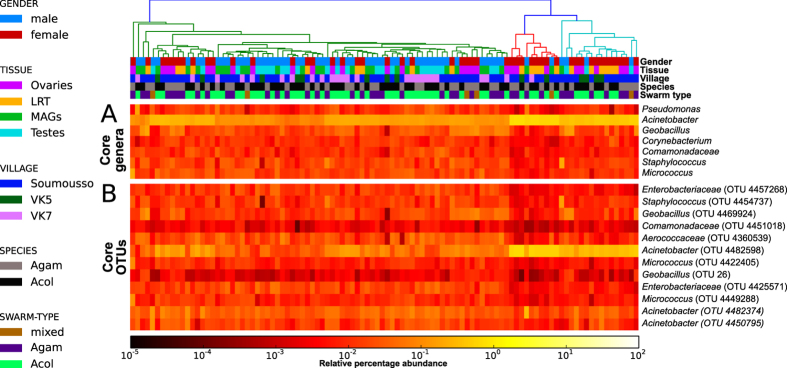
Core reproductive tract microbiome of *An. gambiae* and *An. coluzzii*. (**A**) Genera from both Gram-positive (*Staphylococcus, Corynebacterium, Geobacillus, Micrococcus*) and Gram-negative (*Acinetobacter, Pseudomonas*) bacteria constitute the core microbiome of the *A. gambiae* (Agam) and *A. coluzzii* (Acol) male and female reproductive tissues collected from three villages (VK5, VK7 and Soumousso). (**B**) Different core operational taxonomic units (OTUs) are present within the same genus, suggesting that a consistent species-level microbial diversity characterizes the reproductive tract microbiome. Gender, tissues (Ovaries and Lower Reproductive Tract for females, Testes and Male Accessory Glands for males), villages (Soumousso, VK5 and VK7), species (*An. gambiae*: Agam; and *An. coluzzii:* Acol) and swarm types (individual species or mixed) are color-coded as described in the legend on the left of the figure.

Despite this large intra-sample variability, we identified a highly conserved core microbiome present in all samples and comprising OTUs spanning seven different bacterial genera with variable levels of abundance (Fig. 2). *Acinetobacter*, and specifically OTU 4482598, was the quantitatively dominant microorganism in the majority of the samples (avg. 16.3% s.d. 17.8%, min 0.4%, max 73.3%). This OTU matched the corresponding fragment of several distinct *Acinetobacter lwoffii* sequences stored in the public repositories, although some identical matches were also detected for other closely related species and unnamed organisms in the same genus. Other OTUs present in the core microbiome included *Pseudomonas* and *Staphylococcus*, genera containing species ubiquitous to a large number of environmental and host-associated habitats. *Acinetobacter, Pseudomonas* and *Staphylococcus* have also been detected at lower percentages in the midgut of *An. gambiae* females^11^ and other anophelines^34^. Two OTUs assigned to the *Enterobacteriaceae* were also present in all samples; the 16S rRNA sequence for bacteria in this family can be inconclusive at the genus level, but several organisms in this clade have been reported to colonize the *Anopheles* gut^11^,^35^ with potential interaction with *Plasmodium*^11^,^14^,^36^. *Corynebacterium* was also identified in all samples. As in previous sequencing studies with similar detection sensitivity^11^ this genus was only occasionally found in the *Anopheles* midgut, our finding possibly reflects specificity for reproductive tissues or for our geographical cohort.

We found little evidence of qualitative or quantitative differences in microbial diversity between *An. gambiae* and *An. coluzzii*. Some potentially discriminatory (*p*-value < 0.01) microorganisms including members of *Geobacillus*, *Bacillus*, *Desemzia*, *Oscillospira* and *Burkholderia* were more abundant in *An. gambiae*, while some OTUs in the *Corynebacterium*, *Phycicoccus*, *Proteiniclasticum*, *Nesterenkonia*, *Macrococcus* were more associated with *An. coluzzii* (0.01 ≤ *p*-value < 0.02). None of these OTUs, however, were significantly differentiated between species (*p*-value > 0.05) following correction for multiple hypotheses testing.

**Figure 3:**
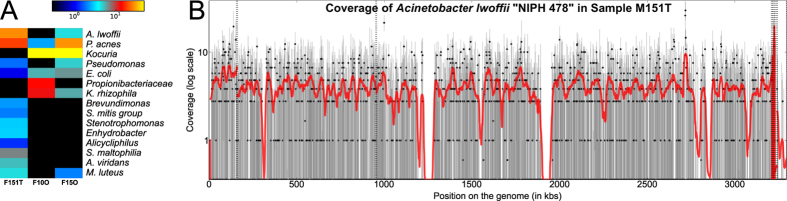
Whole-metagenome shotgun sequencing of testes and ovaries highlights members of the microbiome at the species level. (**A**) Species level abundances as estimated by MetaPhlAn highlight the presence of *Acinetobacter lwoffi, Propionibacterium acnes, Kocuria, Pseudomonas, Kocuria rhizophila* and *Micrococcus luteus* in at least two of the three samples. The other organisms present in one sample only are *Streptococcus mitis*, *Stenotrophomonas maltophilia* and *Aereococcus vididans*. (**B**) Mapping of the metagenomic sample with highest *A. lwoffii* abundance against the genetically closest genome in the species (NIPH 478) highlights a consistent coverage of the large majority of the genes.

Whole-metagenome shotgun sequencing (WGS)^37^ of three *An. coluzzii* reproductive tissues confirmed our results and identified *Acinetobacter lwoffii* as the species in the *Acinetobacter* genus previously detected by 16S rRNA sequencing (Fig. 3A,B). Our *A. lwoffii* reads provided almost complete genome coverage and placed the *Anopheles* strain close to the previously published NIPH 478 (Fig. 3B). *Escherichia coli* was the prevalent microorganism in the *Enterobacteriaceae* family, and *Propionibacterium acnes* were identified in all three samples. We observed lower intra-sample diversity than in the 16S rRNA dataset likely due to the reduced sensitivity achievable by WGS, especially given the high fraction of host DNA contamination. This may explain the absence of *Acinetobacter* in one of three samples sequenced (Fig. 3A).

### Female and male reproductive tracts harbor quantitatively distinct microbial populations

**Figure 4:**
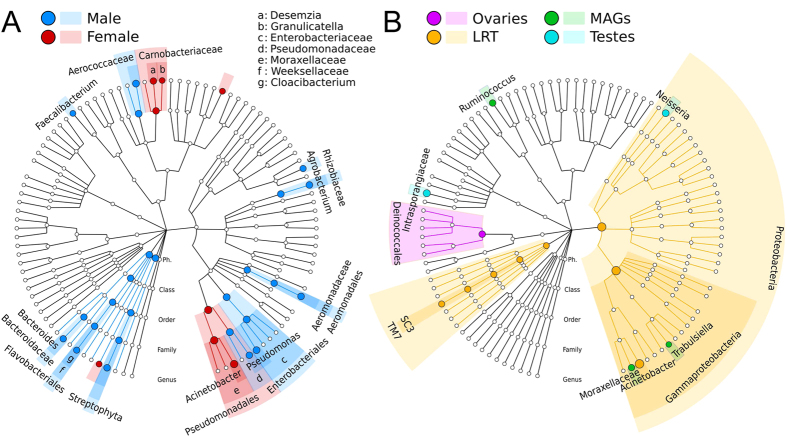
Microbial gender- and tissue-specific biomarkers identified for *An. gambiae* and *An. coluzzi*. (**A**) Hierarchical taxonomic plot of gender-specific microbial biomarkers (LEfSe)^38^ displayed using GraPhlAn^63^. (**B**) Hierarchical taxonomic plot highlighting tissue-specific biomarkers (four-class comparison).

While core bacteria were common to female and male reproductive organs, their quantitative distribution differed between genders. *Acinetobacter* (OTU 4482598 and several other OTUs) drove the sample clustering and were consistently more abundant in the female reproductive tract using the linear discriminant effect size tool, LEfSe^38^ (uncorrected *p* <  1e-5, Fig. 4A). In contrast, *Enterobacteriaceae* and *Aerococcaceae* OTUs were significantly higher in male reproductive tissues (p <  0.01). When expanding this analysis to non-core OTUs, LEfSe detected additional microbial biomarkers associated with female (*Desemzia* and *Granulicatella*) or male (*Agrobacterium*, *Pseudomonas*, *Bacteroides*, and *Cloacibacterium*) reproductive tissues (Fig. 4A).

Tissue-enriched microbial clades were found in each of the four reproductive organs (Fig. 4B). The LRT, in particular, was enriched in bacteria from the phylum Proteobacteria (order Gammaproteobacteria) and in *Acinetobacter* and environmental uncultivable bacteria from the SC3 and TM7 candidate phyla clades. We also identified organisms with evidence of niche adaptation to the MAGs (*Ruminococcus* and *Trabulsiella*, an *Enterobacteraceae* that resembles *Salmonella*), testes (*Neisseria*, a colonizers of mucosal surfaces of many organisms, as well as members of the *Intrasporangiaceae* family), and ovaries (order Deinococcales). The finding of tissue-specific tropism is in line with results from other hosts^39^ including humans^40^. Future studies will be needed to determine whether these microorganisms exert a functional role in modulating the biochemical properties of mosquito reproductive tissues.

An average of 46 (s.d. 10) distinct OTUs with a relative abundance higher than 0.1% were shared between the reproductive tissues of each sex (Supplementary Fig. 3).

### The microbiome diversity in the cohort is enriched by highly variable and sample-specific taxa

**Figure 5:**
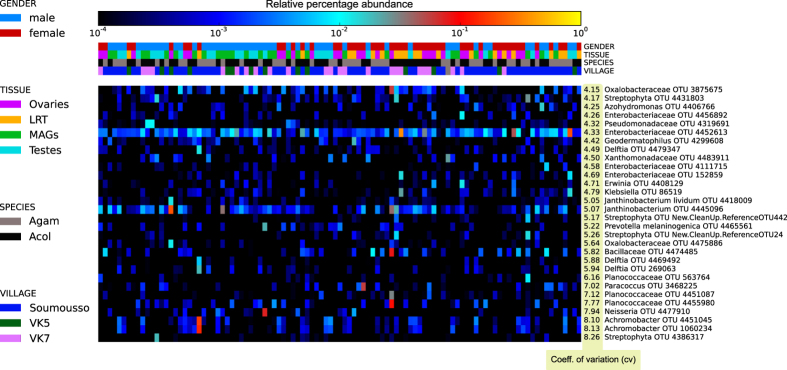
The most variable taxa in the *An. gambiae* and *An. coluzzii* reproductive tract microbiomes. The 30 OTUs with highest score are reported here after computing the coefficient of variation for all the OTUs in our dataset. Each value in the heatmap represents the relative abundance of an OTU in a sample.

Even in the presence of a large core microbiome, we observed high alpha diversity (i.e. intra-sample diversity) due to the presence of many bacteria with partial prevalence but occasional high abundance. Many OTUs showed a large coefficient of variation across samples (Fig. 5), including members of *Enterobacteriaceae*, *Pseudomonadaceae*, *Achromobacter*, and *Deftia*. These highly variable OTUs were not statistically correlated with any of the metadata available (gender, species, tissue, village), which may suggest they are not strictly required for normal micro-ecology and host-microbiome homeostasis, and may result from local environmental acquisition events, specific amplification in favorable conditions, or horizontal transmission.

Some highly abundant microorganisms were found only in a very small fraction of samples. This group included endosymbionts (see below) and other genera such as *Leuconostoc*, a lactic acid-producing coccus, *Arcobacter*, *Ruminococcus* and *Lautropia*, that may be considered opportunistic colonizers (Supplementary Table 4). Given their ability to occasionally invade the reproductive tracts at high relative abundances, these microorganisms might be potentially used for microbiome perturbation strategies aimed at interfering with the physiological host-microbiome homeostasis.

**Figure 6:**
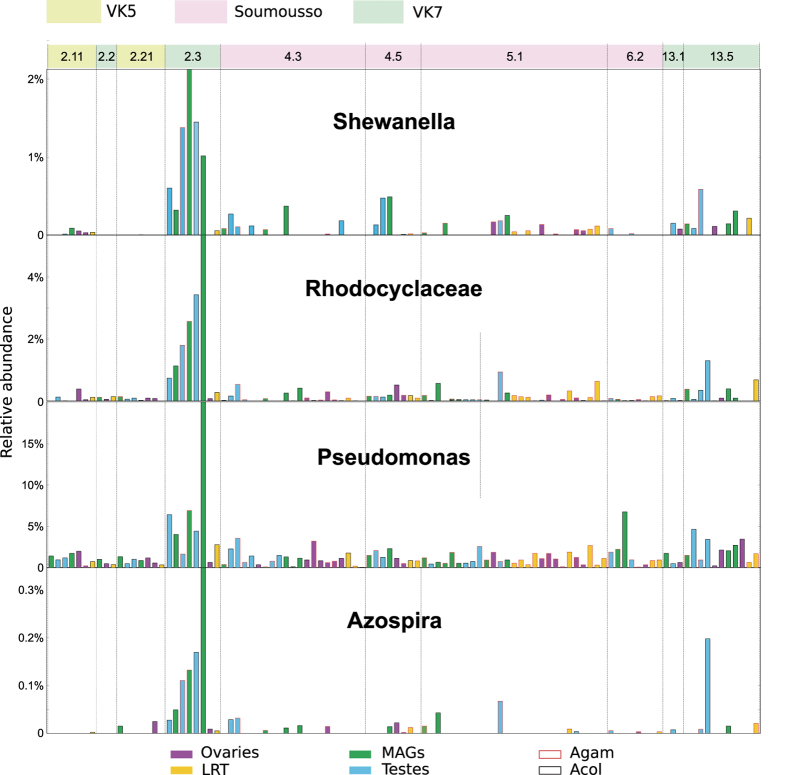
Relative abundance plot for microbial clades strongly associated with males from a specific swarm (swarm 2.3). Bar plots represent the relative abundance (indicated on the Y-axis) of *Shewanella*, Rhodocyclaceae, *Pseudomonas*, and *Azospira* in mosquito samples from three villages (VK5, VK7 and Soumousso, indicated by different color codes. VK5: green; VK7: blue; Soumousso: pink). Each swarm is identified by a numerical code (top bar). Swarm location is provided in Fig. 1. Reproductive tissues collected from male and female individuals from each swarm are represented by color-coded bars. Ovaries: pink; Lower Reproductive Tract (LRT): yellow; Male Accessory Glands (MAGs): green; Testes: blue. Species are indicated by a different bar outline color (*An. gambiae*: red; *An. coluzzii:* black). *Shewanella*, Rhodocyclaceae, *Pseudomonas*, and *Azospira* were highly enriched in male tissues (both MAGs and testes) from a specific swarm (swarm 2.3) from the VK7 village. Please note that some tissues collected from females from swarm 2.3 failed the 16S rRNA sequencing. A detailed list of tissues sequenced for each swarm is provided in Supplementary Table 2.

We found an enrichment in specific bacteria in some swarm locations. Three males collected in the VK7 village from the same mating swarm (swarm 2.3, adjacent to a rice paddy, Fig. 1) showed a highly significant enrichment in *Shewanella*, *Rhodocyclacea*, *Pseudomonas* and *Azospira* in both testes and MAGs (Fig. 6). These bacteria were detected at much lower abundance in females mated to these males, as well as in males and females from swarm locations at the other end of the VK7 village or in the other villages. *Shewanella*, *Rhodocyclacea* and *Pseudomonas* share some genetic similarities with each other and are often found in water, including rice fields, while some *Azospira* genera are root bacteria with metal bioactivity. As the reproductive microbiome of adults is largely shaped during larval development, it is likely that local environmental factors have favored the proliferation of these microbes in the rice fields surrounding the swarm location. These findings suggest the hypothesis that males from the same larval breeding sites may tend to swarm together. Genetic analyses will be needed to test the hypothesis of a possible degree of kinship within male swarms.

### Endosymbionts with potential relevance for malaria control are present at partial prevalence

Among the organisms present at high relative abundance in a small fraction of the samples were the endosymbionts *Asaia*^23^, *Rickettsia*, *Spiroplasma*^41^, *Thorsellia anophelis*^42^ and the previously characterized *Wolbachia*^20^ (Table 1). Bacterial endosymbionts which live within the tissues or cells of their host are widespread across arthropods species^43^ and generally persist by maternal transmission. *Asaia* were detected in only 5% of the collected samples (Table 1), in contrast with prior reports on laboratory colonies and field populations^39^. We did, however, detect members of the *Acetobacteraceae* family in 72% of the samples, with OTUs assigned to the genera *Acetobacter*, *Gluconobacter*, *Roseococcus*, and *Roseomonas*, all acetic acid-producing bacteria known to colonize a wide range of insects^39^. *Spiroplasma*, an endosymbiont that can colonize germline cells and manipulate host reproductive output^44^, was identified in the LRT of an *An. gambiae* female and, at lower abundances, in the testes of an *An. coluzzii* male (Supplementary Fig. 4). To our knowledge, this is the first time that *Spiroplasma* has been identified in the *Anopheles* reproductive tract. In addition, the gammaproteobacterium *Thorsellia anopheles* was detected in ovaries and testes from both species (Table 1). *T. anophelis* was first identified in the midgut of *An. arabiensis* in central Kenya^45^ and appears to have adapted to the female anopheline midgut by utilizing blood and tolerating the alkaline conditions present in this tissue^42^. *Thorsellia* is closely related to the genus *Arsenophonus*^42^ which comprises endosymbionts of arthropod species^46^.

## Discussion

Our analysis of the reproductive tract microbiomes of two major malaria vectors reveals the presence of a large core microbiome spanning seven bacterial genera (12 OTUs) shared by all tissues (Fig. 2). When relaxing the definition of core microbiome by considering organisms present in at least 90% of the individuals, the number of common reproductive tract colonizers expands to 54 OTUs. Given the number of samples and their diversity in terms of host species (*An. gambiae* and *An. coluzzii*), tissues (MAGs, testes, LRT, ovaries), gender, and geographical origin (three villages and several distinct swarms), our findings suggest that the core reproductive microbiome identified here may be shared by other anopheline populations, with broad implications for using these bacteria to stop malaria transmission by the mosquito vector.

Our original hypothesis of different reproductive microbiomes populating *An. gambiae* and *An. coluzzii* was not supported by our data. Although surprising given the vastly diverse larval habitats occupied by these two species, this result may be due to the depth of 16S rRNA analysis, and therefore we cannot conclusively rule out a role of the reproductive microbiome in mediating adaptation to different ecological niches or in shaping specific adult behaviors including mating. Our analyses, however, unexpectedly revealed a number of bacteria (*Shewanella*, *Rhodocyclacea*, *Pseudomonas* and *Azospira*) selectively enriched in males from the same swarm, a mixed swarm containing both *An. gambiae* and *An. coluzzii* males. These two mosquito species mate in swarms that are formed at dusk in specific locations which are conserved during the same season and even across different years^47^. How mosquitoes recognize these specific locations is not known but choice is thought to depend on how sites are attractive to females, for instance for blood feeding opportunities^27^. Swarm 2.3 from which those males were captured is next to a rice field (Fig. 1), presumably representing the nearest breeding sites available for larval development. Apart from swarm 2.2, which is situated in close proximity to swarm 2.3 but from which only one male tissue could be sequenced (Fig. 6, Supplementary Table 2), all other swarms from VK7 are located in the opposite end of the village and may be associated with different breeding sites (Fig. 1). The four bacterial genera enriched in males from swarm 2.3 can all grow in rice paddies, and it is plausible that the specific microenvironment of neighboring rice paddies specifically favored their proliferation. As bacteria are likely to colonize reproductive tissues during larval development, our observation of males (but not females) from the same swarm sharing specific bacteria suggests these males originated from the same breeding site, indicating a possible degree of kinship between males in swarms. Females are thought to fly over longer distances than males^48^, potentially explaining why the same bacteria were not detected in the reproductive tract of the single female that we analyzed from that swarm. An alternative explanation is bacterial tropism for male rather than female reproductive tissues. Further studies are needed to test this hypothesis and to further unravel the mating ecology of these mosquitoes.

A number of endosymbionts were identified that may have reproductive interactions with their mosquito hosts. We identified *Spiroplasma* bacteria in the testes and ovaries of two individuals. Endosymbiotic *Spiroplasma* infect approximately 5–15% of all insect species^43^,^49^, living primarily in the hemolymph and gut^41^ from where they can be horizontally transmitted^50^. *Spiroplasma* infections have been detected in *An. gambiae*^45^, *An. funestus*^45^ and several species of the *Aedes* and *Culex* genera^51^,^52^,^53^,^54^, but this may be the first time they have been identified in the reproductive tract of malaria mosquitoes. These bacteria can persist in their female host through two main strategies: either providing an indirect fitness advantage to females by inducing male killing^55^ or by directly protecting the host against natural pathogens^44^,^56^. Therefore, *Spiroplasma* infections possess two key characteristics that might be exploited for disease control, namely an ability to spread through mosquito populations and a protective function against pathogens.

Contrary to higher estimates previously obtained using targeted PCR investigations^39^, we detected *Asaia* only in 5% of samples. This apparent inconsistency could be explained by a potential positive bias of the PCR primers used in the 16S rRNA sequencing for the entire *Acetobacteraceae* family that comprises *Asaia*. Many OTUs could only be assigned at the family level, which suggests either the presence of unknown genera in this family or the existence of organisms belonging to subclades of known genera (including *Asaia*) that are still uncharacterized in the 16S rRNA databases. We hypothesize that bacteria from *Asaia* and very closely related organisms are indeed common colonizers of the reproductive tracts of *An. gambiae* and *An. coluzzii*, and that the current characterization and sampling of *Asaia* is not capturing the overall in-field diversity of this genus. As *Asaia* is also identified in the mosquito gut and has been proposed for paratransgenesis strategies for its ability to be paternally and maternally transmitted^21^,^22^,^23^, it will be important to accurately determine its prevalence in natural *Anopheles* populations.

The finding of a core reproductive microbiome is highly relevant for blocking *Plasmodium* infections. Paratransgenesis approaches have been proposed to control the transmission of malaria parasites through the use of bacteria producing anti-*Plasmodium* agents^17^. The strength of these approaches depends on factors including the ability to re-engineer bacterial genomes, the infectivity of the bacteria for mosquitoes, and the fitness costs associated with infection that affect their ability to spread through insect populations. Our study identified several candidates in the core microbiome that could be used for this purpose. Of particular interest is the widespread presence of *A. lwoffii*, a relatively well-characterized, cultivable and non-pathogenic bacterium commonly found in the rhizosphere that has already been proposed as a biocontrol agent for plant protection and has been found in the midgut of *An. gambiae*^11^ and other anophelines^34^. The strain we have identified in field *Anopheles* populations is highly abundant in the reproductive organs and may have potential for paratransgenic approaches. At a time when widespread resistance to all classes of insecticides currently used for mosquito control is threatening the success of our best weapons against malaria^57^, these and other core bacteria may furnish novel and desperately needed tools for the control of *Plasmodium* transmission in mosquito populations.

## Materials and Methods

### Mosquito collections and rRNA sequencing

Mosquito samples were collected during August-September 2011 in three villages near Bobo-Dioulasso, Burkina Faso. Two villages in Vallée du Kou, VK5 (11°23′N; 4°24′W), which is delimited by rice fields, and VK7 (11°24′N; 04°24′W) surrounded by savannah to the North and rice fields to the South, where *An. coluzzii* (M form) is more abundant^58^. The village of Soumousso (11°00′N; 4°02′W) is characterized by savannah and by temporary breeding sites that are more favorable to *An. gambiae* (S form)^58^. We collected 30 mating couples *in copula* from different swarms and we dissected the male and female reproductive tracts 1 to 3 h later. Reproductive tissues included the testes and male accessory glands (MAGs) for males, and the ovaries and lower reproductive tract (LRT, which comprises the atrium, the spermatheca and the parovarium) for females.

For *An. gambiae* and *An. coluzzii* species determination, DNA from legs was extracted by incubating an individual leg in 40 μl of grinding buffer (10 mM Tris-HCl pH 8.2, 1 mM EDTA, 25 mM NaCl) with 0.2 mg/ml proteinase K for 45 min at 37 °C, then 5 min at 95 °C. One μl of each DNA extract was then used for the locus S200 × 6.1 PCR amplification. DNA extraction and 16S rRNA sequencing were performed as described in^20^.

Genomic DNA was extracted using DNeasy (Qiagen) and subjected to *16S* rRNA amplifications similar to^59^. Briefly, primers comprising a sample barcode sequence and the Illumina adapters were used to allow directional sequencing covering of the variable region V4 (15F: 5′GTGCCAGCMGCCGCGGTAA-3′; and 806R: 5′GGACTACHVGGGTWTCTAAT-3′). PCR reactions included 10 μl of DNA template diluted 1:50, 10 μl of HotMasterMix with the HotMaster Taq DNA Polymerase (5 Prime), and 5 μl of primer mix (2 μM of each primer). The cycling conditions were: 94 °C for 3 min, 30 cycles at 94 °C for 45 sec, 50  °C for 60 sec, 72 °C for 5 min, and a final 72 °C for 10 min. DNA amplification was quantified and pooled in equimolar concentrations on the Pippin Prep (Sage Sciences, Beverly, MA) with size selected (375–425 bp) to minimize non-specific amplification products from host DNA. A final library size and quantification was done on an Agilent Bioanalyzer 2100 DNA 1000 chips (Agilent Technologies, Santa Clara, CA). Sequencing was performed on the Illumina MiSeq v2 platform, and paired-end reads of 175b in length in each direction were generated. The overlapping paired-end reads were stitched together (approximately 97 bp overlap), and size selected to reduce non-specific amplification products from host DNA (225–275 bp).

### 16S rRNA and whole-genome shotgun sequencing and analysis

MiSeq sequencing of the V4 variable region of the 16S rRNA gene generated a total of 2.32 M reads (average 22,560, s.d. 14,471 reads per sample) from 102 high-quality samples (18 samples were removed because of technical failures). This dataset was pre-processed and analyzed with the Qiime pipeline^32^ for taxonomic composition, alpha diversity, and beta diversity analysis^32^,^33^, as previously described^20^, to characterize the microbiome structure, composition, and variability.

Three samples were also subject to deep shotgun metagenomic sequencing (Illumina HiSeq 2000 with NexteraXT Library preparation) generating a total of 1,29 M reads (avg 431 M, s.d. 22 M, Supplementary Table S3), that were processed for host DNA removal with BowTie2^60^ against the *Anopheles* genomes, species-level taxonomic profiling with MetaPhlAn^33^,^61^, coverage analysis with SAMTools^62^, and biomarker discovery with LEfSe^38^. Cladograms and phylogenetic trees were displayed using GraPhlAn^63^.

## Additional Information

**Accession codes:** Sequence reads for the 16S-rRNA amplicon samples have been deposited in the NCBI Sequence Read Archive (SRA) under accession code SRR610826. The three shotgun metagenomic samples are available in the NCBI Sequence Read Archive (SRA) under accession codes *PRJNA244534 and SRX1631720*.

**How to cite this article**: Segata, N. *et al.* The reproductive tracts of two malaria vectors are populated by a core microbiome and by gender- and swarm-enriched microbial biomarkers. *Sci. Rep.* **6**, 24207; doi: 10.1038/srep24207 (2016).

## Supplementary Material

Supplementary Information

## Figures and Tables

**Table 1:** Abundance and prevalence of endosymbiotic microorganisms identified in the *An. gambiae* and *An. coluzzii* reproductive microbiome.

Most precise taxonomicclade	Maximumabundance	Samples withthis OTU	Species	Tissue
*Thorsellia anophelis*	15.35%	8 [7.92%]	3 *An. gambiae*,5 *An. coluzzii*	5 MAGs,2 Testes,1 Ovaries
*Asaia*	7.16%	5 [4.95%]	2 *An. gambiae*,3 *An. coluzzii*	3 MAGs,1 Testes,1 Ovaries
*Spiroplasma*	7.74%	2 [1.98%]	1 *An. gambiae*,1 *An. coluzzii*	1 Testes, 1 LRT
*Wolbachia*	19.26%	1 [0.99%]	1 *An. coluzzii*	1 Testes
*Rickettsia*	3.23%	1 [0.99%]	1 *An. gambiae*	1 MAGs
