# Supplementary material for: The reproductive tracts of two malaria vectors are populated by a core microbiome and by gender- and swarm-enriched microbial biomarkers
Source: Sci Rep. 2016 Apr 18;6:24207. doi: 10.1038/srep24207 (PMC4834568; doi:10.1038/srep24207)
Supplement: Supplementary Information [file srep24207-s1.pdf]

The reproductive tracts of two malaria vectors are populated by a core microbiome and by gender- and swarm-enriched microbial biomarkers

SUBTITLE: The natural reproductive microbiome of *Anopheles* mating couples

Nicola Segata[1, #], Francesco Baldini[2,3,4, #], Julien Pompon[5,6,7], Wendy S. Garrett[2,8,9,10], Duy Tin Truong[1], Roch K. Dabiré[11], Abdoulaye Diabaté[11], Elena A. Levashina[5,12, *] and Flaminia Catteruccia[2,4,*]

Supplementary material

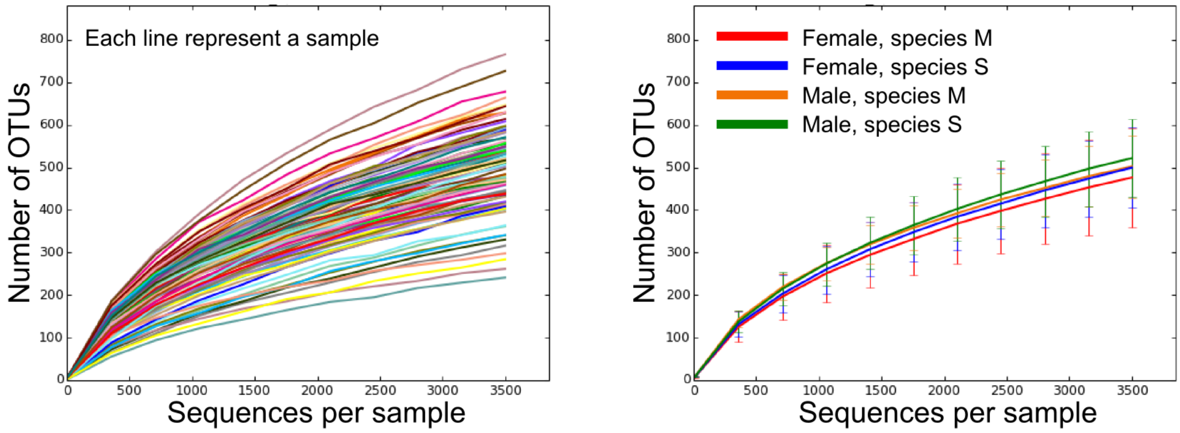

Supplementary Figure S1. Intra sample diversity (alpha-diversity) of the reproductive microbiome of male and female *A. gambiae* and *A. coluzzii*. The microbiota diversity varies among samples and does not show consistent patterns with respect to external metadata including species, gender, tissue, and village. The diversity measure adopted (number of OTUs) is plotted at increasing subsampling size in order to avoid biases due to uneven sequencing depths.

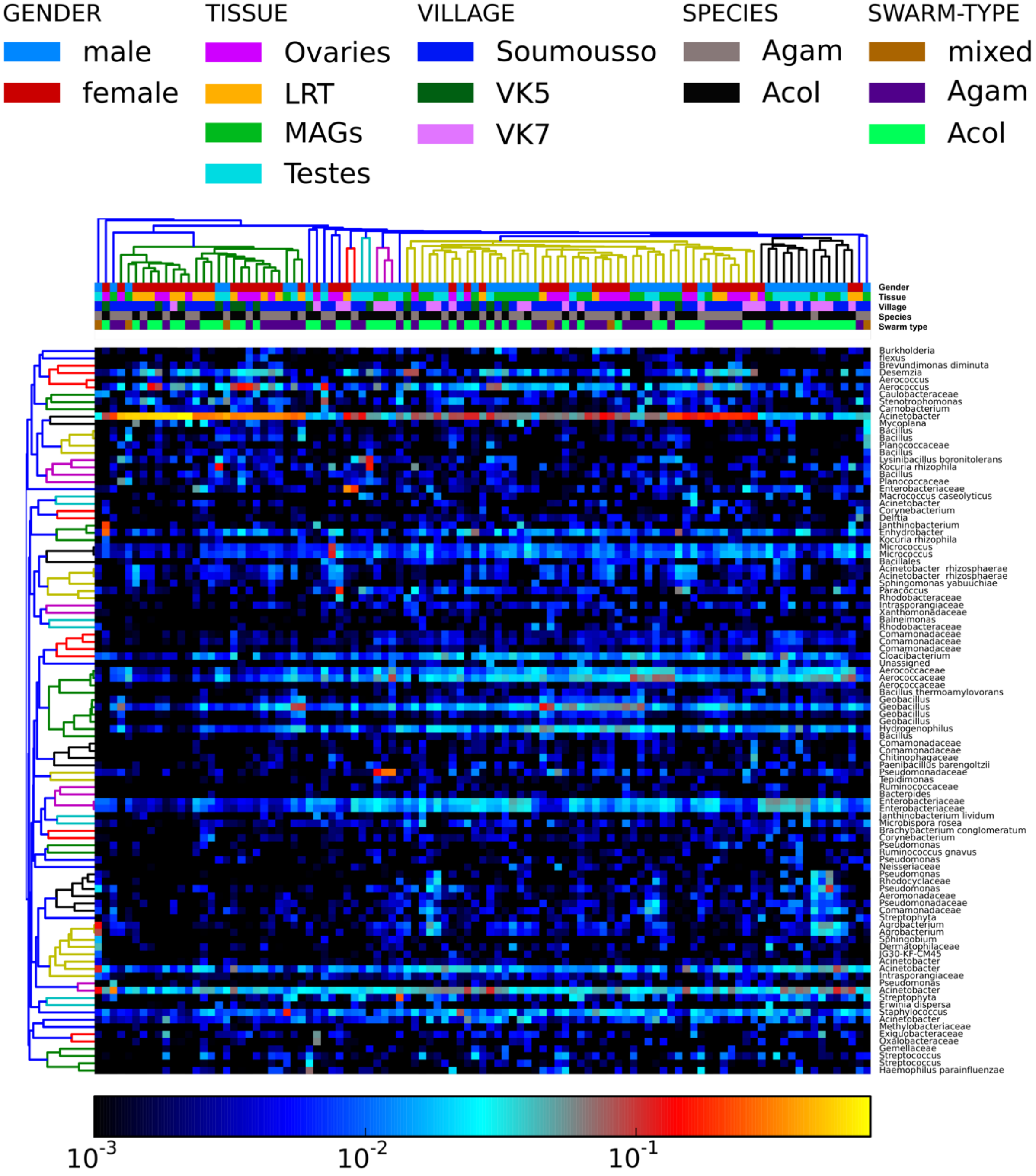

Supplementary Figure S2. The 100 most abundant OTUs in the reproductive microbiome. The 100 most abundant OTUs are reported with the corresponding taxonomic assignments shown on the right. The OTU ranking was computed based on the 99th percentile of each OTU' abundance pattern.

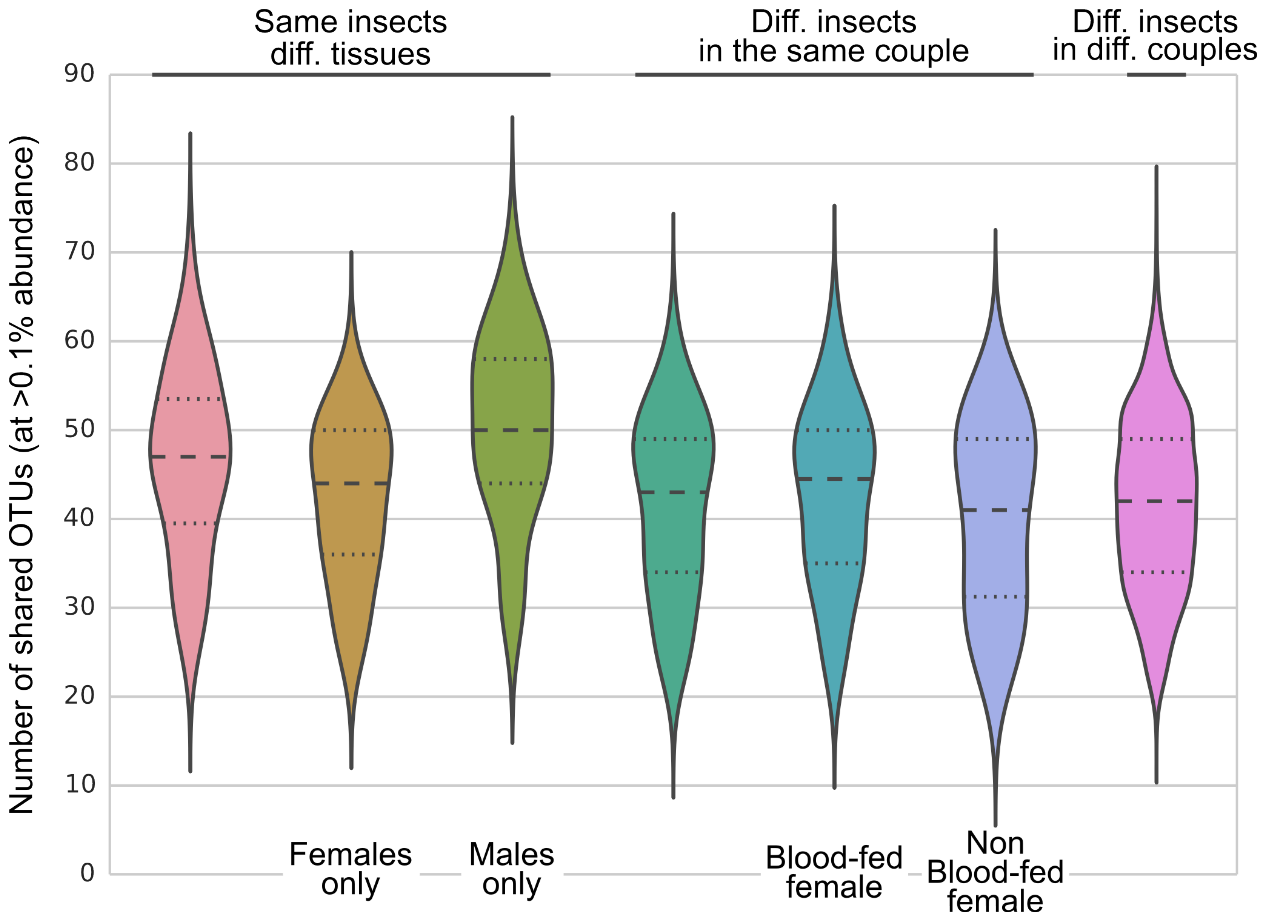

Supplementary Figure 3. Distribution of the number of shared highly abundant (>0.1% relative abundance) among different set of samples. From left to right, the seven violin plots (in which dashed lines represent the median and interquartile ranges) report the distribution of the number of shared OTUs among different reproductive tissues of the same insect (overall and divided by gender), among the two components of the mating couple (overall and divided by blood feeding status), and among insects from unrelated couples.

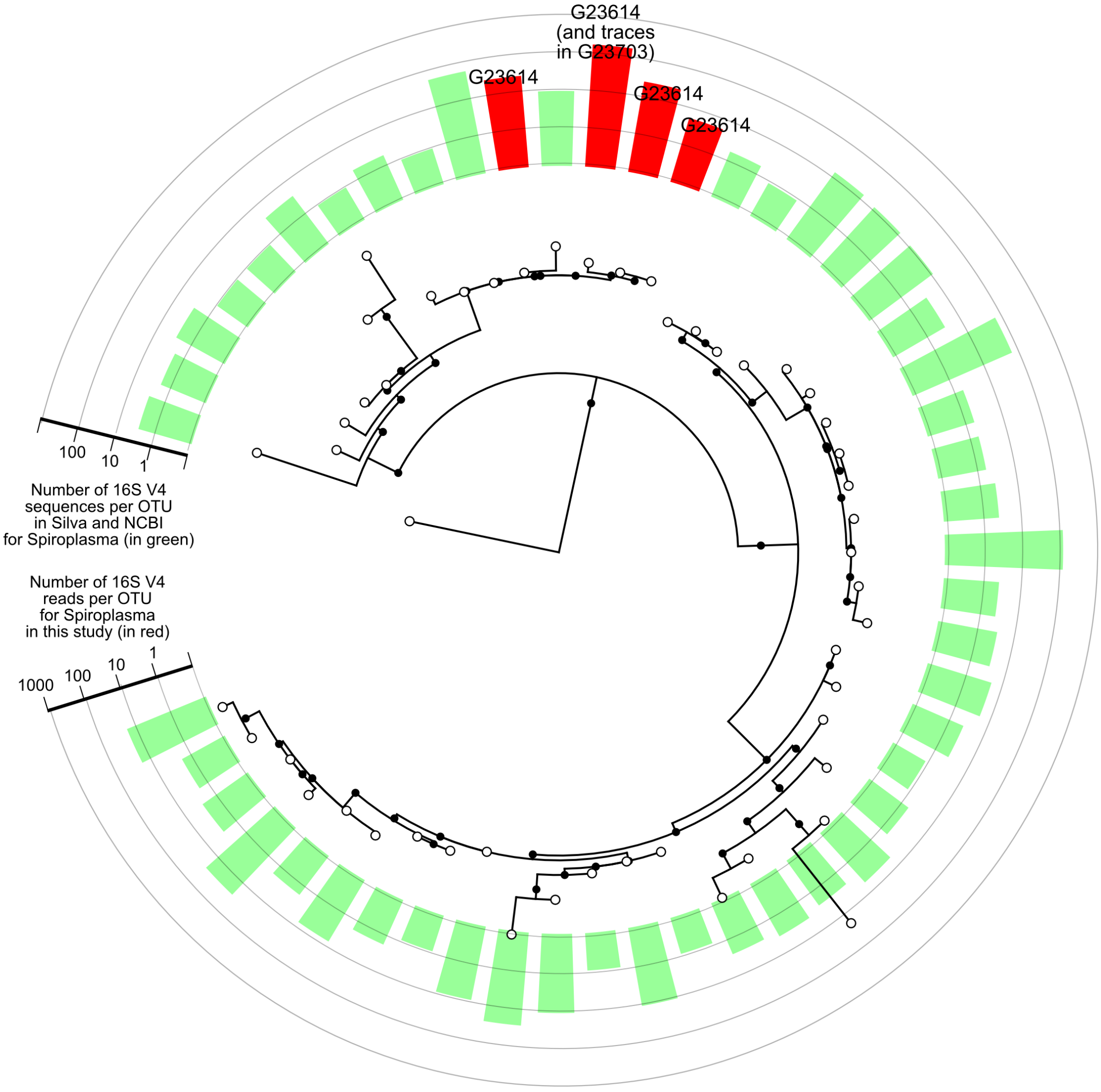

Supplementary Figure S4. ***Spiroplasma* consistently clusters in a subtree of the 16S rRNA phylogenetic tree of this genus**. The tree was built using V4 16S rRNA fragments from *Spiroplasma* reference sequences available in public repositories (NCBI and SILVA) and from the sequences obtained in this study. The sequences are clustered at 99% identity and the cardinality of each OTU obtained is reported in a logarithmic scale as a bar chart external to the tree (green for reference sequences and red for the new sequences).

Supplementary Table S1. Summary of mating couple collection from 3 villages. Each individual was genotyped (*An. gambiae* and *An. coluzzii*). From each swarm where mating couples were collected, 20 swarming males were also collected and genotyped to determine the predominant species of the swarm.

**Village**	**Species**	**Couples**	**Couple type**	**Swarms**	**Swarm type**
Soumousso	24 *An. gambiae*, 12 *An. coluzzii*	18	11 *An. gambiae*, 5 *An. coluzzii*, 2 mixed	11	8 *An. gambiae*, 2 *An. coluzzii*, 1 mixed
VK5	8 *An. coluzzii*	4	4 *An. coluzzii*	2	2 *An. coluzzii*
VK7	2 *An. gambiae*, 14 *An. coluzzii*	8	6 *An. coluzzii*, 2 mixed	4	4 *An. coluzzii*
Total	26 *An. gambiae*, 24 *An. coluzzii*	30	11 *An. gambiae*, 15 *An. coluzzii*, 4 mixed	17	8 *An. gambiae*, 8 *An. coluzzii*, 1 mixed

Supplementary Table S2. List of all sample tissues successfully sequenced. Each sample corresponds to a reproductive tissue from an individual, which is identified by its ID corresponding to the gender and a numerical code for each mating couple collected. Swarm locations can be identified in the village map in Figure 1.

ID	Tissue	Village	Species	Swarm location
84_male	MAGs	Soumousso	*An. gambiae*	5.1
84_female	LRT	Soumousso	*An. gambiae*	5.1
84_female	Ovaries	Soumousso	*An. gambiae*	5.1
82_male	Testes	Soumousso	*An. gambiae*	5.1
82_female	LRT	Soumousso	*An. gambiae*	5.1
82_female	Ovaries	Soumousso	*An. gambiae*	5.1
81_male	MAGs	Soumousso	*An. gambiae*	5.1
81_male	Testes	Soumousso	*An. gambiae*	5.1
81_female	LRT	Soumousso	*An. gambiae*	5.1
81_female	Ovaries	Soumousso	*An. gambiae*	5.1
73_male	MAGs	Soumousso	*An. gambiae*	5.1
73_male	Testes	Soumousso	*An. gambiae*	5.1
73_female	LRT	Soumousso	*An. gambiae*	5.1
73_female	Ovaries	Soumousso	*An. gambiae*	5.1
66_male	MAGs	Soumousso	*An. gambiae*	4.3
66_male	Testes	Soumousso	*An. gambiae*	4.3
66_female	Ovaries	Soumousso	*An. gambiae*	4.3
65_male	MAGs	Soumousso	*An. gambiae*	4.3
65_male	Testes	Soumousso	*An. gambiae*	4.3
65_female	Ovaries	Soumousso	*An. gambiae*	4.3
56_male	MAGs	Soumousso	*An. coluzzii*	4.5
56_male	Testes	Soumousso	*An. coluzzii*	4.5
56_female	LRT	Soumousso	*An. coluzzii*	4.5
56_female	Ovaries	Soumousso	*An. coluzzii*	4.5
54_male	MAGs	Soumousso	*An. coluzzii*	4.3
54_male	Testes	Soumousso	*An. coluzzii*	4.3
54_female	LRT	Soumousso	*An. coluzzii*	4.3
54_female	Ovaries	Soumousso	*An. coluzzii*	4.3
53_male	MAGs	Soumousso	*An. coluzzii*	4.3
53_male	Testes	Soumousso	*An. coluzzii*	4.3
53_female	LRT	Soumousso	*An. coluzzii*	4.3
53_female	Ovaries	Soumousso	*An. coluzzii*	4.3
52_male	Testes	Soumousso	*An. coluzzii*	4.3
52_female	LRT	Soumousso	*An. coluzzii*	4.3
52_female	Ovaries	Soumousso	*An. coluzzii*	4.3
51_male	MAGs	Soumousso	*An. coluzzii*	5.1
51_male	Testes	Soumousso	*An. coluzzii*	5.1
43_male	MAGs	Soumousso	*An. gambiae*	6.2
43_male	Testes	Soumousso	*An. gambiae*	6.2
43_female	LRT	Soumousso	*An. gambiae*	6.2
43_female	Ovaries	Soumousso	*An. gambiae*	6.2
42_male	MAGs	Soumousso	*An. gambiae*	6.2
42_male	Testes	Soumousso	*An. gambiae*	6.2
42_female	LRT	Soumousso	*An. gambiae*	6.2

42_female	Ovaries	Soumousso	*An. gambiae*	6.2
38_male	MAGs	Soumousso	*An. gambiae*	4.5
38_male	Testes	Soumousso	*An. gambiae*	4.5
38_female	LRT	Soumousso	*An. gambiae*	4.5
38_female	Ovaries	Soumousso	*An. gambiae*	4.5
36_male	MAGs	Soumousso	*An. gambiae*	4.3
36_male	Testes	Soumousso	*An. gambiae*	4.3
36_female	Ovaries	Soumousso	*An. gambiae*	4.3
36_female	Ovaries	Soumousso	*An. gambiae*	4.3
34_male	MAGs	Soumousso	*An. coluzzii*	5.1
34_male	Testes	Soumousso	*An. coluzzii*	5.1
34_female	LRT	Soumousso	*An. gambiae*	5.1
34_female	LRT	Soumousso	*An. gambiae*	5.1
34_female	Ovaries	Soumousso	*An. gambiae*	5.1
33_male	MAGs	Soumousso	*An. gambiae*	5.1
33_female	LRT	Soumousso	*An. gambiae*	5.1
33_female	Ovaries	Soumousso	*An. gambiae*	5.1
31_male	MAGs	Soumousso	*An. coluzzii*	5.1
31_female	Ovaries	Soumousso	*An. gambiae*	5.1
130_male	MAGs	VK5	*An. coluzzii*	2.21
130_male	Testes	VK5	*An. coluzzii*	2.21
130_female	Ovaries	VK5	*An. coluzzii*	2.21
125_male	MAGs	VK5	*An. coluzzii*	2.21
125_male	Testes	VK5	*An. coluzzii*	2.21
125_female	LRT	VK5	*An. coluzzii*	2.21
125_female	Ovaries	VK5	*An. coluzzii*	2.21
122_male	MAGs	VK5	*An. coluzzii*	2.11
122_male	Testes	VK5	*An. coluzzii*	2.11
122_female	LRT	VK5	*An. coluzzii*	2.11
122_female	Ovaries	VK5	*An. coluzzii*	2.11
121_male	MAGs	VK5	*An. coluzzii*	2.11
121_male	Testes	VK5	*An. coluzzii*	2.11
121_female	Ovaries	VK5	*An. coluzzii*	2.11
9_male	MAGs	VK7	*An. coluzzii*	13.5
9_male	Testes	VK7	*An. coluzzii*	13.5
9_female	LRT	VK7	*An. coluzzii*	13.5
9_female	Ovaries	VK7	*An. coluzzii*	13.5
6_male	Testes	VK7	*An. gambiae*	13.5
6_male	MAGs	VK7	*An. gambiae*	13.5
6_female	Ovaries	VK7	*An. coluzzii*	13.5
20_male	MAGs	VK7	*An. coluzzii*	2.3
20_male	Testes	VK7	*An. coluzzii*	2.3
20_female	Ovaries	VK7	*An. coluzzii*	2.3
19_male	MAGs	VK7	*An. gambiae*	2.3
19_male	Testes	VK7	*An. gambiae*	2.3
18_male	MAGs	VK7	*An. coluzzii*	2.3
18_male	Testes	VK7	*An. coluzzii*	2.3
18_female	LRT	VK7	*An. coluzzii*	2.3
111_male	MAGs	VK7	*An. coluzzii*	2.2
111_female	LRT	VK7	*An. coluzzii*	2.2
111_female	Ovaries	VK7	*An. coluzzii*	2.2
11_male	MAGs	VK7	*An. coluzzii*	13.5
11_male	Testes	VK7	*An. coluzzii*	13.5

11_female	LRT	VK7	*An. coluzzii*	13.5
11_female	Ovaries	VK7	*An. coluzzii*	13.5
104_male	MAGs	VK7	*An. coluzzii*	13.1
104_male	Testes	VK7	*An. coluzzii*	13.1
104_female	Ovaries	VK7	*An. coluzzii*	13.1

Supplementary Table S3. The number of reads in three shotgun sequencing samples.

**Sample**	**Total number of reads**	**Number of reads after quality control and host DNA removal**
F15O	411,813,322	77,121,291
M151T	427,348,200	75,974,350
F10O	455,105,864	79,716,497

Supplementary Table S4. List of OTUs present in a small fraction of samples (<17%) but present in some samples at high abundance (>3%). Endosymbionts are highlighted in bold.

Most precise taxonomic clade for each OTU	Maximum abundance in a sample	Number of samples in which the OTU is present (%)
Arcobacter	18.76%	16 (15.5%)
Leuconostoc	3.65%	15 (14.6%)
Leuconostoc	3.42%	13 (12.6%)
Leuconostoc	13.49%	13 (12.6%)
Thorsellia anophelis	15.35%	8 (7.8%)
Lautropia	3.69%	8 (7.8%)
Oribacterium	6.50%	7 (6.8%)
Rhizobiaceae	5.58%	7 (6.8%)
Ruminococcus	4.41%	6 (5.8%)
Asaia	7.16%	4 (3.9%)
Porphyromonas endodontalis	3.63%	3 (2.9%)
Spiroplasma	7.74%	2 (1.9%)
Carnobacterium	4.35%	2 (1.9%)
Wolbachia	19.26%	1 (1%)
Weeksellaceae	9.26%	1 (1%)
Rickettsia	3.23%	1 (1%)
Euglenozoa	3.13%	1 (1%)
